# *Foxa1* and *Foxa2* Are Required for Formation of the Intervertebral Discs

**DOI:** 10.1371/journal.pone.0055528

**Published:** 2013-01-31

**Authors:** Jennifer A. Maier, YinTing Lo, Brian D. Harfe

**Affiliations:** Molecular Genetics and Microbiology and the Genetics Institute, University of Florida, College of Medicine, Gainesville, Florida, United States of America; Indiana University School of Medicine, United States of America

## Abstract

The intervertebral disc (IVD) is composed of 3 main structures, the collagenous annulus fibrosus (AF), which surrounds the gel-like nucleus pulposus (NP), and hyaline cartilage endplates, which are attached to the vertebral bodies. An IVD is located between each vertebral body. Degeneration of the IVD is thought to be a major cause of back pain, a potentially chronic condition for which there exist few effective treatments. The NP forms from the embryonic notochord. *Foxa1* and *Foxa2*, transcription factors in the forkhead box family, are expressed early during notochord development. However, embryonic lethality and the absence of the notochord in *Foxa2* null mice have precluded the study of potential roles these genes may play during IVD formation. Using a conditional *Foxa2* allele in conjunction with a tamoxifen-inducible *Cre* allele (*ShhcreER^T2^*), we removed *Foxa2* from the notochord of E7.5 mice null for *Foxa1*. *Foxa1^−/−^*;*Foxa2^c/c^*;*ShhcreER^T2^* double mutant animals had a severely deformed nucleus pulposus, an increase in cell death in the tail, decreased hedgehog signaling, defects in the notochord sheath, and aberrant dorsal-ventral patterning of the neural tube. Embryos lacking only *Foxa1* or *Foxa2* from the notochord were indistinguishable from control animals, demonstrating a functional redundancy for these genes in IVD formation. In addition, we provide *in vivo* genetic evidence that *Foxa* genes are required for activation of *Shh* in the notochord.

## Introduction

Three main structures compose the intervertebral disc (IVD); the annulus fibrosus (AF), the nucleus pulposus (NP), and endplates (EP). A disc is sandwiched between each of the vertebrae along the axial skeleton of vertebrates and allows for flexibility while providing a major support to the body. The NP is a watery gel-like substance containing proteoglycans [1], [2]. The NP is formed from the embryonic notochord [3] and it is encircled by the AF, a structure comprised of many concentric rings of collagen fibers [1]. The AF is derived from the sclerotome [4], a compartment of the somite. Hyaline cartilage endplates bound the disc superiorly and inferiorly and attach each disc to the vertebrae [1], [2]. It is through the endplates that the disc, which is avascular and is not innervated by nerves, obtains its nutrients via diffusion [1], [2], [5].

During the aging process numerous changes occur to the disc and these are collectively referred to as “disc degeneration” [6]–[8]. These changes include the transition of the NP from a watery gel-like substance to a more fibrous material, the accumulation of small tears in the AF [2], [7], [9] and ossification and fracturing of the endplates [10], [11]. All of these changes alter the nutrition and structural integrity of the disc, and thus the axial column. In some patients, the NP can herniate out of the AF while in other patients the disc is observed to become compressed resulting in a decrease in the distance between adjacent vertebrae. These changes can result in abnormal pressure being placed on the numerous nerves present in adjacent tissue resulting in severe pain. Back pain, while common, has few effective treatments and is an expensive ailment in terms of medical costs and lost productivity [12], [13]. Current treatments address the pain and not the underlying causes, which is frequently disc degeneration. Surgery, and complete or partial disc replacement are options for patients suffering chronic pain but often have have high rates of failure and unacceptable side effects for patients [2], [9]. It is hoped that by studying the proper formation of the disc and factors that contribute to its degeneration, we can develop novel, effective strategies for disc repair/regeneration.

The notochord is a rod-like structure found in all chordate embryos, and is surrounded by a sheath (the “notochordal” sheath), which is composed of extracellular matrix proteins [14]. During embryonic development the notochord secretes sonic hedgehog (SHH) protein (reviewed in [15]) and serves as a vital signaling center in the chordate embryo [16]. In some species, the notochord also serves as the main structural support for the embryo on which the rest of the body plan is built [16]. The notochord itself forms from the mammalian organizer, or node. This is located at the anterior end of the primitive streak in a mouse embryo.

Cells of the trunk notochord are formed from cells that migrate through the node during gastrulation [17]. The node is capable of inducing a second axis when transplanted into a host embryo [18]. The tail notochord was thought to form from the regression of the node posteriorly, though more recent fatemaps of node cells have suggested that instead, these cells migrate through the node posteriorly to form the tail notochord [19] [20]. The mouse notochord has 3 separate origins; anterior cells that are not node-derived, trunk notochord that forms from cells of the node that undergo convergent extension, and tail notochord which has been proposed to form from cells that have migrated out of the node [19]. The tail bud forms at the end of gastrulation and eventually undergoes secondary neurulation to form the notochord and neural tube in the posterior embryo [21], [22].

*Foxa* genes, formerly termed HNF3 (hepatocyte nuclear factors), were identified by their ability to bind to liver-specific enhancers [23], [24]. In mice, the *Foxa* subfamily has three members, *Foxa1*, *Foxa2* and *Foxa3* [25]. They have been extensively studied in several tissues [26]–[29] and are required for embryonic development and post-natal life [30]. *Foxa1* and *Foxa2* are expressed in the notochord [25], [31]–[33] and (Figure S1; File S1); *Foxa3* is not reported to be found in this structure and was not investigated in the current study.

*Foxa1* null mice contained a normal notochord and intervertebral discs but died within days of birth as a result of hypoglycemia and dehydration [26]. *Foxa2* null mice died during early embryogenesis and lacked a node and notochord in addition to containing abnormalities in all germ layers [34], [35]. Due to the absence of a node (and thus notochord), potential roles for *Foxa2* in NP formation have not been identified. In this study, we investigated disc formation using a *Foxa2* conditional floxed mouse [36] in combination with a *Foxa1* null allele [26]. Both alleles have previously been used to characterize the development of the liver, lung, brain, and other tissues [27], [29], [37]. To remove *Foxa2* from the notochord, the tamoxifen-inducible *ShhcreER^T2^* allele was used [38]. Removal of both *Foxa1* and *Foxa2* from the notochord resulted in severe defects in the formation of the axial skeleton and aberrant dorsal-ventral patterning of the neural tube. The individual removal of either *Foxa1* or *Foxa2* resulted in normal disc formation indicating that the *Foxa* genes are functionally redundant in the mouse notochord. Defects in the notochord of double mutants were observed by E9.5, well before the disc starts to form at E12.5. A molecular analysis revealed that *Foxa* gene expression in the notochord was required for expression of *Shh*.

## Materials and Methods

### Animal Care, Generation of Embryos and Genotyping

All mice were raised in accordance with University of Florida Institutional Animal Care and Use Committee Guidelines (UF IACUC) under specific pathogen-free (SPF) conditions. All procedures were approved by the UF IACUC. Animals containing the *Foxa1* null allele (*Foxa1^−/−^*) and *Foxa2* conditional allele (*Foxa2^c/c^*) were originally generated by the Kaestner laboratory [26], [36]. These animals were mated to mice containing the tamoxifen-inducible *ShhcreER^T2^* [38] and *R26R* [39] alleles to create mice in which *Foxa1* and *Foxa2* could be deleted from the notochord (Fo*xa1^−/−^*;*Foxa2^c/c^*;*ShhCreER^T2^*;*R26R*). In this report, these animals are referred to as “double mutants”. Inclusion of the *R26R* allele allowed for fate mapping of mutant cells. *R26R* was not present in every animal analyzed. Timed matings were obtained by setting up a cross in the late afternoon; the following morning the female was monitored for the presence of a semen plug, and was by convention determined to have mated at 12:00 am the night before (Day 0.5 when plug is found). Animals homozygous for the *Foxa2* conditional allele were phenotypically normal. Weanlings were ear-tagged and genotyped using the tip of the tail. To genotype embryos, extra-embryonic membranes were removed during dissection and lysed in 25 mM NaOH at 95°C for at least 20 minutes. Samples were neutralized with an equal volume of 40 mM TrisHCl and 1–2 µL DNA used in PCR reactions. Genotyping was performed as previously described [26], [36], [38], [39]. At least three mutant animals were examined for each experiment. Control animals were either *Foxa1^+/+^*;*Foxa2^c/c^* or *Foxa1^+/−^*;*Foxa2^c/c^*. Mice were maintained on a mixed genetic background.

### RNA *in situ* hybridization

Anti-sense probes for *in situ* hybridization were made using a linear template and an *in vitro* RNA synthesis reaction with Roche reagents. RNA contained a digoxigenin (DIG) label was used. For whole-mount RNA *in situs*, embryos were dissected in phosphate buffered saline (PBS), fixed overnight in 4% paraformaldehyde (PFA), and then changed to 1% PFA or dehydrated in methanol then bleached with 6% hydrogen peroxide. Embryos were processed for *in situs* or stored in a −20°C freezer until ready to use. *In situ* hybridization was performed using standard methods. Briefly, day 1 involved treatment with proteinase K, fixation, and overnight incubation with the antisense probe. On day 2 unbound probe was washed off the sample and antibody against the DIG label was added, day 3 was washes, and on day 4 embryos were developed in BM Purple (Roche) or a mixture of BCIP (5-bromo-4-chloro-3′-indolyphosphate p-toluidine salt) and NBT (nitro blue tetrazolium); both are substrates of alkaline phosphatase, which is conjugated to the anti-DIG antibody. Embryos were developed in the dark; when they were ready they were washed several times with PBS before being fixed and photographed. For section *in situs*, embryos were dissected in diethyl pyrocarbonate (DEPC)-treated PBS with RNAseZap (Ambion). After overnight fixing in 4% DEPC PFA, embryos were changed to a 30% DEPC sucrose/PBS solution overnight. Finally, embryos were incubated in a 1∶1 mix of 30% Sucrose∶OCT (Optimal Cutting Temperature, Sakura Tissue-Tek) for two hours, then embedded in 100% OCT and stored at −80°C until use. Embryos were sectioned on a Leica cryostat at 12–14 µm and stored at −80°C. Before proceeding with *in situs*, slides were dried and sections traced with a hydrophobic pen. All solutions used on Day 1 were DEPC treated and 10% dextran sulphate was added to the prehybe solution. Otherwise procedure was similar to whole-mount *in situ* hybridization. Slides were coverslipped with parafilm for overnight incubations. The protocol was based on previously published *in situ* protocols [40], [41] and resources from the Tabin lab website (Harvard University). An unabridged *in situ* protocol is in the File S1. Probes used have been described previously: *Shh* [*42*], *T (Brachyury)* [*43*], *Noto* [*44*], *Tbx18* [*45*], *Pax1* [*46*], *Pax3* [*47*], *Nkx2.2* [*48*], *Nkx6.1* [*48*], and *Ptch1* [*49*]. *Foxa1* and *Foxa2* probes were made from IMAGE clones from Open Biosystems: *Foxa1* (4911145) and *Foxa2* (6488102).

### LacZ staining

Embryos were collected and fixed in 0.2% PFA overnight. The next day embryos positive for the *R26R* and *ShhcreER^T2^* alleles were rinsed 3×10 minutes in LacZ rinse buffer (0.1M sodium phosphate [pH 7.4], 0.1% sodium deoxycholate, 2 mM MgCl_2_, 0.2% NP-40) before being put in LacZ staining solution (1 mg/mL X-gal in DMF, 5 mM K_3_Fe(CN)_6_, 5 mM K_4_Fe(CN)_6_) overnight in the dark at room temperature. Reactions were stopped the following day by 3×10 minute washes in PBS. Embryos were photographed and then fixed overnight in 4% PFA.

### Histology

Fixed tissue was dehydrated in an ethanol series before being embedded in paraffin by standard methods and sectioned at 7 µm on a microtome. LacZ-stained tissue was sectioned at 10 µm. Slides were dewaxed in xylenes for 10 minutes followed by rehydration in an ethanol series for 10 minutes each. Slides were washed for 15 minutes in Alcian blue stain (Sigma, pH 2.5), followed by 10 minutes in tap water. Picrosirius red (PolyScientific) staining was done for 45 minutes and then an acid wash followed (0.025% acetic acid in deionized water), followed by de-staining for 1 minute each in 95% and 100% ethanol, then 10 minutes in xylenes. Slides were mounted with Permount (Fisher Scientific) and coverslipped. For LacZ-stained slides, they were dewaxed and rehydrated as described and then counterstained with Nuclear Fast Red for 5 minutes, and then de-stained, xylene washed and coverslipped as above.

### Nuclear Fast Red & Alcian blue staining

Embryos embedded in OCT were serially sectioned at 10 µm on a cryostat. Slides was washed in Alcian blue for 15 minutes, followed by 10 minutes in tap water, 5 minutes in Nuclear Fast Red, and then dipped in deionized water before being mounted with Glycergel (DAKO).

### Immunohistochemistry

Tissue was fixed in 0.2% PFA overnight and embedded in paraffin. Paraffin slides were dewaxed and rehydrated as described above, and then antigen retrieval was performed in a conventional microwave at 70% power for 15 minutes in 10 mM sodium citrate buffer +0.1% Tween20. Slides were allowed to cool for 30 minutes and then were blocked for 30 min to 2 hours with 10% donkey serum in PBST. Primary antibody for FOXA2 (AbCam, ab5074) was added at 1∶75 and LAMININ (DAKO, Z0097) 1∶200 in 10% donkey serum, coverslipped with parafilm, and left overnight at 4°C. The next day slides were washed with PBST several times, blocked again for 30 minutes in 10% donkey serum/PBST, and secondary antibody was added at 1∶300 for 1 hour (AlexaFluor 568 donkey anti-goat IgG, Invitrogen and DyLight 488 donkey anti-rabbit IgG, Jackson ImmunoResearch). After washing with PBST and counterstaining with DAPI, slides were mounted with DAKO Fluorescent mounting media and coverslipped.

### LysoTracker assay

Embryos were dissected into PBS that had been warmed to 37°C. LysoTracker Red reagent (Invitrogen) was added (25 µL to 5 mL warm PBS) and 500 µL was added to each embryo. Embryos were incubated at 37°C in the dark for 30 minutes and then rinsed several times with PBS before being fixed in 4% PFA overnight. The next day, embryos were dehydrated in a methanol series for at least 30 minutes each in 25%, 50%, 75%, and 100% methanol. Embryos were stored at −20°C in the dark until photographed.

## Results

### Removal of *Foxa1* and *Foxa2* in the mouse notochord

**Figure 1:**
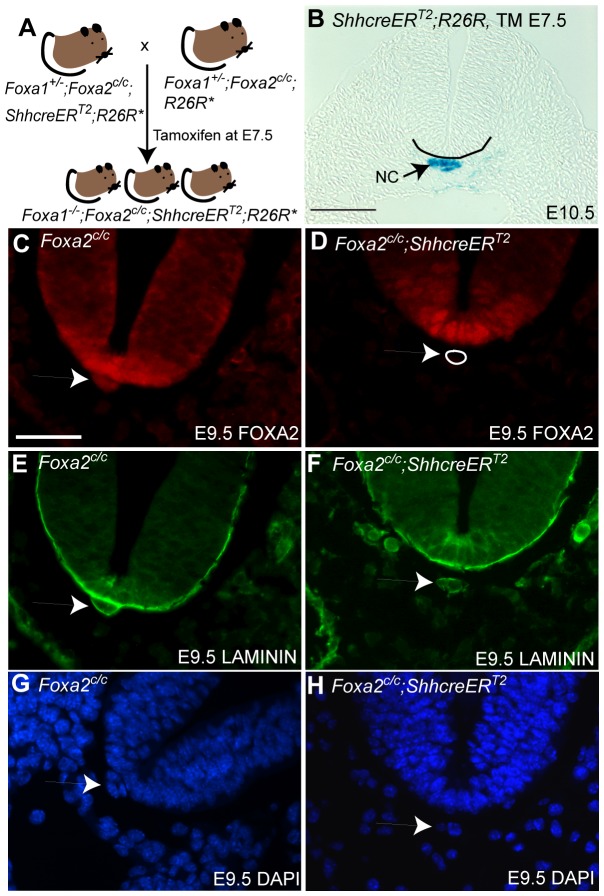
Strategy to remove FOXA2 from the mouse notochord. A: Mice heterozygous for the *Foxa1* null allele (*Foxa1^+/−^*), homozygous for the *Foxa2* floxed conditional allele (noted as *Foxa2^c/c^* in this report) and that contained the *ShhcreER^T2^* allele were crossed to *Foxa1^+/−^*;*Foxa2^c/c^* mice to create double knockouts. The pregnant dam was given tamoxifen by oral gavage (4 mg) in corn oil when embryos were E7.5. * indicates that the *R26R* allele was crossed into some animals for fate-mapping. B: Exposure of E7.5 embryos to tamoxifen that contained the *ShhcreER^T2^* and CRE-inducible *R26R LacZ* reporter resulted in reporter expression in the notochord (NC) but not the floorplate. The *ShhcreER^T2^* allele is not active in the floor plate at E7.5. Scale bar is 100 µm. The ventral neural tube is outlined in black for clarity. C–F: Confirmation of removal of *Foxa2^c/c^* from the notochord using a FOXA2-specific antibody. C and D: FOXA2 protein was present in the notochord and floorplate of control embryos (C) but absent from the notochord of *Foxa2^c/c^*;*ShhcreER^T2^* embryos (D; the notochord is outlined in white). E and F: The presence of a notochord in *Foxa2^c/c^*;*ShhcreER^T2^* embryos was confirmed by staining for Laminin protein. Dissociation of the notochord from the neural tube in D and F is likely a result of tissue processing. G and H: DAPI staining of a similar E9.5 section to show nuclear staining. White arrows in C–H denote the notochord. C–H: Scale bar is 50 µm.

The *Foxa1* null allele and the *Foxa2* conditional floxed allele have been described previously [26], [36]. Mice containing these alleles were crossed to animals containing the *ShhcreER^T2^* allele [38] to generate *Foxa1^+/−^*;*Foxa2^c/c^*;*ShhcreER^T2^* mice. These animals were phenotypically normal since the *ShhcreER^T2^* allele only produces CRE protein in the presence of tamoxifen. Double mutant embryos were obtained by crossing *Foxa1^+/−^*;*Foxa2^c/c^*;*ShhcreER^T2^* males to *Foxa1^+/−^*;*Foxa2^c/^* ^c^ females (diagrammed in Figure 1A). Tamoxifen was administered at E7.5 by oral gavage to remove the *Foxa2* conditional allele in embryos that were homozygous for the *Foxa1* null allele. In this report, mice that were null for *Foxa1* and had *Foxa2* removed in *Shh*-expressing cells are referred to as “double mutants”.

*Cre* activity in the notochord, upon administration of tamoxifen at E7.5, was confirmed using the CRE-inducible *R26R* reporter allele [39]; (Figure 1B). *Shh* is normally expressed in many tissues besides the notochord, including the floorplate [42]. At the stage embryos were exposed to tamoxifen (E7.5), *Shh* has not been reported to be expressed in the floorplate. Consistent with these observations, CRE recombination of the *R26R* reporter was observed in the notochord but not the floorplate in embryos that had been given a single dose of tamoxifen at E7.5. These data are consistent with our previous published report in which we demonstrated that tamoxifen administration at E7.5 drives Cre expression in the notochord but not the floor plate [50].

To confirm the removal of *Foxa2* from the notochord in tamoxifen-treated embryos, immunohistochemistry for FOXA2 was performed using an antibody raised to a part of the protein that is deleted upon CRE-induced recombination [51]. FOXA2 protein was found in the floorplate and notochord of E9.5 control embryos that had been treated with tamoxifen at E7.5 but lacked the *ShhcreER^T2^* allele (Figure 1C). In *Foxa2^c/c^*;*ShhcreER^T2^* embryos, FOXA2 was present in the floorplate but was absent from the notochord (Figure 1D). Immunohistochemistry was performed for Laminin protein, which among other structures, outlined the notochord demonstrating that despite a lack of FOXA2 staining in the notochord that this tissue was still present in mutant animals (Figure 1E,F). Morphology of the notochord and neural tube area was also visualized with DAPI (Figure 1G,H).

### *Foxa1*;*Foxa2* double knockouts have severely deformed nuclei pulposi

To determine if *Foxa1*;*Foxa2* double mutants contained defects in the intervertebral discs, late stage embryos were analyzed (*Foxa1* null mice die just after birth [26]). Double mutants were observed to contain a shortened tail that frequently lacked any discernable structures such as vertebrae or discs (Figure S2H,I). Vertebral columns of controls and mutants were analyzed using Alcian blue and picrosirius red stains. Picrosirius red stains collagens [52], [53] while Alcian blue stains cartilage and mucopolysaccharides [54].

**Figure 2:**
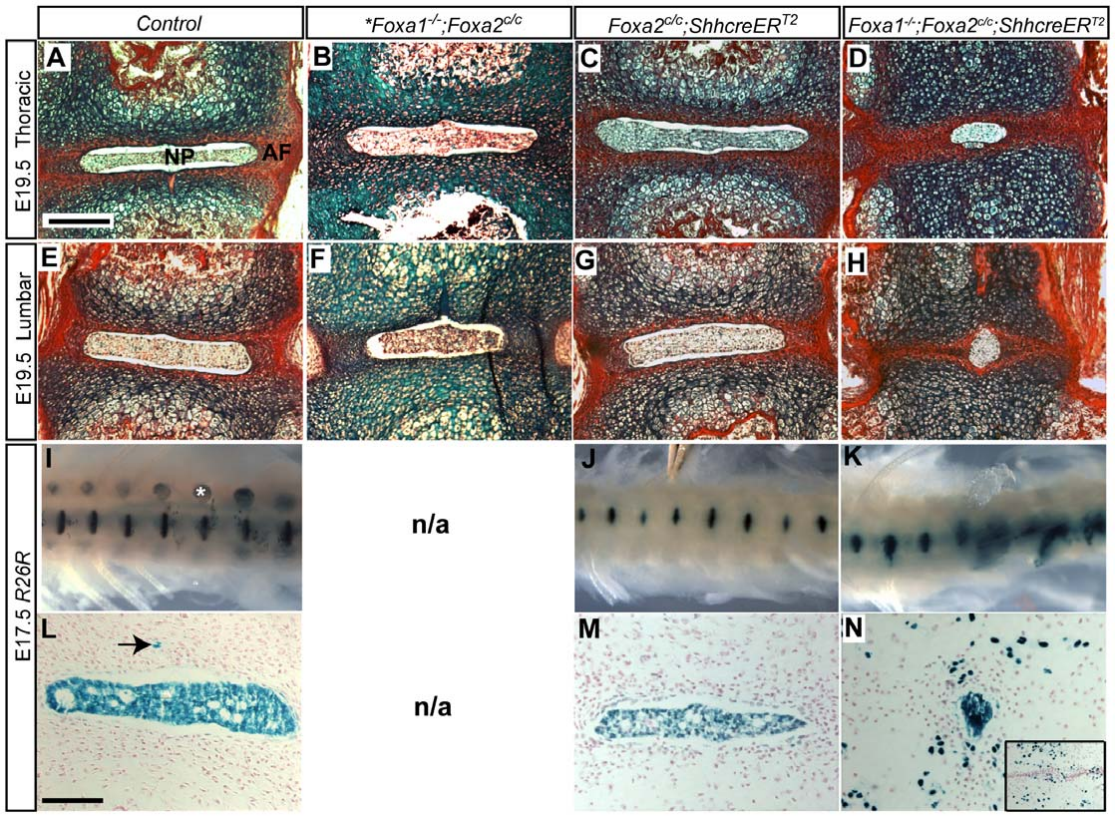
*Foxa1*;*Foxa2* double mutants have severe defects in intervertebral disc formation. A–H: Alcian blue and picrosirius red staining of disc sections. Scale bar is 200 µm. Thoracic sections at E19.5 show a normal sized nucleus pulposus (NP) surrounded by a red-stained annulus fibrosus (AF) in control (A) *Foxa1* null (B) and *Foxa2* notochord knockout (C) embryos. Asterisk in *Foxa1^−/−^*;*Foxa2^c/c^* column denotes that embryo was E17.5. Disc morphology was also normal at the lumbar region in these mice (E, F, G). In *Foxa1*;*Foxa2* double mutants (D and H) nuclei pulposi were abnormal. Nuclei pulposi were observed to be much smaller than nuclei pulposi present in control or individual single mutants. I and J: A fatemap of notochord cells using the *R26R* reporter allele demonstrated proper nuclei pulposi formation in control (I) and *Foxa2* notochord knockouts (J). In contrast, *Foxa1*;*Foxa2* double mutants (K) had severely deformed nuclei pulposi and blue cells were dispersed. Defects were more severe in the posterior. (I) is an embryo of the genotype *Shhgfpcre*;*R26R*. White asterisk marks dorsal root ganglia. The *Shhgfpcre* allele is constitutively active, therefore all *Shh*-expressing cells, including dorsal root ganglia, and their descendants are *LacZ*-positive. All LacZ positive cells in J and K are derived exclusively from the notochord [3]. In our mating it was not possible to obtain a *Foxa1^−/−^* notochord fatemap without also removing the floxed *Foxa2* allele, therefore the *Foxa1^−/−^* fatemap is not shown (N/A = not applicable). L, M and N: Sections of LacZ-stained tissue at the lumbar level. Inset in N shows an additional section outside of the plane of the NP; numerous cells are scattered throughout the disc region and vertebral bodies. L–M: Scale bar is 100 µM. Arrow in L denotes a notochord remnant, which is a notochord cells that does not reside in the NP.

Control animals had fully-formed intervertebral discs with clearly identifiable nuclei pulposi and annuli fibrosi (Figure 2A,E and Figure S2A,E). It has been reported that expression of CRE protein can cause phenotypes in some tissue types [55]. To determine if CRE protein produced from the *ShhcreER^T2^* locus could cause disc defects, *Foxa2^c/c^*;*ShhcreER^T2^* animals were examined in detail. An extensive histological analysis revealed that the vertebral columns from these embryos were indistinguishable from controls (Figure 2C,G and Figure S2C,G). These data also indicated that removal of only *Foxa2* from the notochord was not sufficient to cause a phenotype in the vertebral column. In addition, removal of only *Foxa1* from all cells (*Foxa1^−/−^*;*Foxa2^c/c^* embryos) also resulted in normal NP and vertebrae (Figure 2B,F and Figure S2B,F). For more details, see Supporting Information.

To determine if the lack of a phenotype in individual *Foxa* mutants was a result of compensation by the remaining gene, both *Foxa1* and *Foxa2* were removed from the embryonic notochord (*Foxa1^−/−^*;*Foxa2^c/c^*;*ShhcreER^T2^*). In double mutants the NP was compressed and small (Figure 2D and H). The disc defects were generally more severe posteriorly than anteriorly. Double mutant embryos, had shorter tails (Figure S2I), that frequently lacked disc and vertebral structures (Figure S2H). The ossification centers were also abnormal in the double mutants (Figure S2D) in comparison to control (Figure S2A) and single knockouts (Figure S2B,C). These data suggest that *Foxa1* and *Foxa2* can compensate for each other during formation of the intervertebral discs and vertebrae, consistent with reports in other tissues in which phenotypes were only observed when both genes were removed from the same tissue [27], [29].

### The notochord to nuclei pulposi transition is abnormal in *Foxa1*;*Foxa2* double mutants

Nuclei pulposi form entirely from the embryonic notochord [3]. To determine if the notochord to nuclei pulposi transition was abnormal in *Foxa1*;*Foxa2* double mutants, notochord cells were fate mapped using the *R26R* CRE-inducible reporter allele [39]. The *R26R* reporter allele expresses *LacZ* in all cells in which CRE protein has been expressed. Embryos containing the *ShhcreER^T2^* allele that have been exposed to tamoxifen at E7.5 express *LacZ* in the notochord. Importantly, once cells express *LacZ* they, and all their descendants will express LacZ even if CRE protein is not longer present.

**Figure 3:**
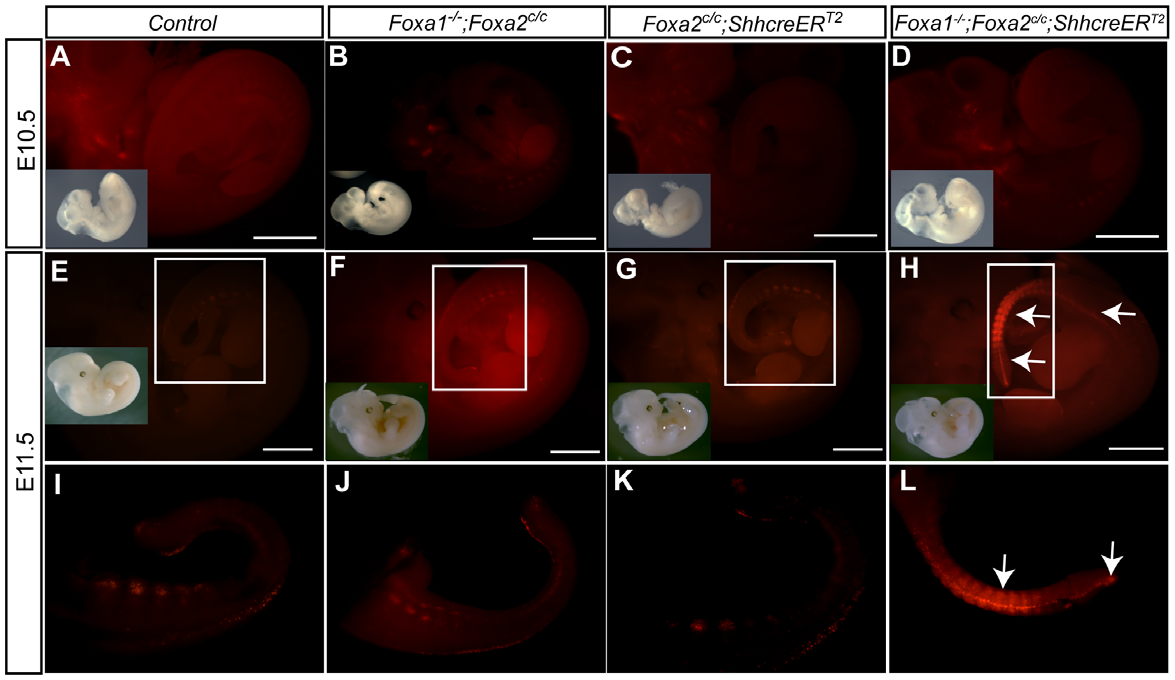
Cell Death is increased in the tail of E11.5 but not E10.5 *Foxa1*;*Foxa2* double mutant embryos. Cell death was assayed using LysoTracker on live embryos at E10.5 and E11.5. A–D. Control (A), *Foxa1* null (B), *Foxa2* notochord knockouts (C), and *Foxa1*;*Foxa2* double knockout embryos (D) had similar amounts of cell death at E10.5. Inset is a brightfield image of an E10.5 embryo shown for reference. Scale bar for the LysoTracker is 1 mm. E–H: At E11.5, control (E and I), *Foxa1* null (F, J) and *Foxa2* notochord knockout (G and K) embryos had LysoTracker positive cells in the somites, dorsal tail, and end of the tail but were indistinguishable from one another. In contrast, double mutant embryos (H and L) had numerous LysoTracker positive cells in the somites and midline of the tail (white arrows). Images I–L are zoomed in areas denoted by the boxes in E–H. The insets in E–H are brightfield images of E11.5 embryos. The scale bar for the LysoTracker images in E–H is 1 mm.

Double mutants containing the *R26R* allele were harvested at E17.5 and treated with β-galactosidase to determine the fate of notochord cells. In control embryos (Figure 2I,L) and embryos lacking *Foxa2* (Figure 2J,M) notochord cells formed nuclei pulposi. In contrast, in double mutant embryos notochord cells were observed to reside throughout the vertebral column (Figure 2K,N). It was not possible to follow the fate of notochord cells in animals from the floxed *Foxa2^c/c^* mating since inclusion of the *Cre* allele would have resulted in deletion of the floxed *Foxa2* allele, thus we used mice containing the *Shhgfpcre* allele which constitutively express *Cre* in all *Shh* descendants including the dorsal root ganglia (Figure 2I, [3]). It was also not possible to obtain a *Foxa1* null sample for LacZ staining due to the construction of our mating scheme (see Figure 1A).

### Cell death occurs in posterior somites and the tail of *Foxa1*;*Foxa2* double mutants

To examine cell death in *Foxa1*;*Foxa2* knockout embryos, LysoTracker Red reagent (Invitrogen) was used. At E10.5 similar levels of cell death were observed in control, single and double mutants (Figure 3A–D). However, at E11.5 *Foxa1*;*Foxa2* double mutants displayed massive cell death in the posterior somites and midline of the tail (Figure 3H,L) compared to control (Figure 3E,I), *Foxa1* null (Figure 3F,J), and *Foxa2* notochord mutant (Figure 3G,K) littermates. This dramatic amount of cell death likely results in the shorter tail found in E17.5 (Figure S2I) and E19.5 double mutants. Due to the presence of ectopic cell death in double mutants beginning at E11.5, all gene expression analysis was performed prior to this embryonic stage.

### *Foxa* expression in the notochord is not required for formation of the sclerotome

**Figure 4:**
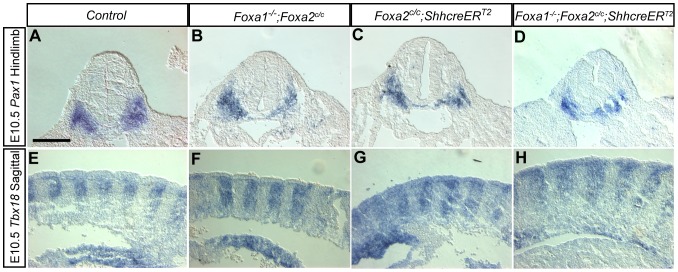
The sclerotome is initially unaffected in *Foxa1*;*Foxa2* double knockouts. A–D: *Pax1* expression at the hindlimb level (transverse sections are shown). *Pax1* is expressed in the sclerotome at E10.5. In controls (A), *Foxa1* nulls (B), *Foxa2* notochord knockouts (C), and double mutants (D) there was no difference in *Pax1* expression. E–H: E10.5 sagittal sections showing *Tbx18* expression at mid-trunk level. *Tbx18* is confined to the anterior sclerotome. No differences in expression of *Tbx18* were observed in single (F and G) and double (H) mutants compared to control embryos (E). Scale bars are 200 µm.

*Pax1* and *Pax9*, two transcription factors expressed in the sclerotome, are involved in the development of the vertebral column. *Pax1*;*Pax9* double mutant mice do not form IVDs and lose the segmental arrangement of vertebrae [56]. In this study we examined *Pax1*, which is expressed in the early sclerotome, using section RNA *in situ* hybridization. *Pax1* was expressed robustly in the sclerotome in the E10.5 forelimb (Figure S3A–D and File S1) and hindlimb levels of all embryos (Figure 4A–D). *Tbx18* maintains the anterior-posterior polarity of the somites, and its loss results in abnormalities of the axial skeleton [57]. *Tbx18*, expressed in the anterior somite [57], [58], was also found to be indistinguishable from control (Figure 4E), single knockout (Figure 4F and G), and *Foxa1*;*Foxa2* double mutants (Figure 4H) at E10.5.

### *Noto* and *T* (*Brachyury*) expression in *Foxa1*;*Foxa2* double mutants

**Figure 5:**
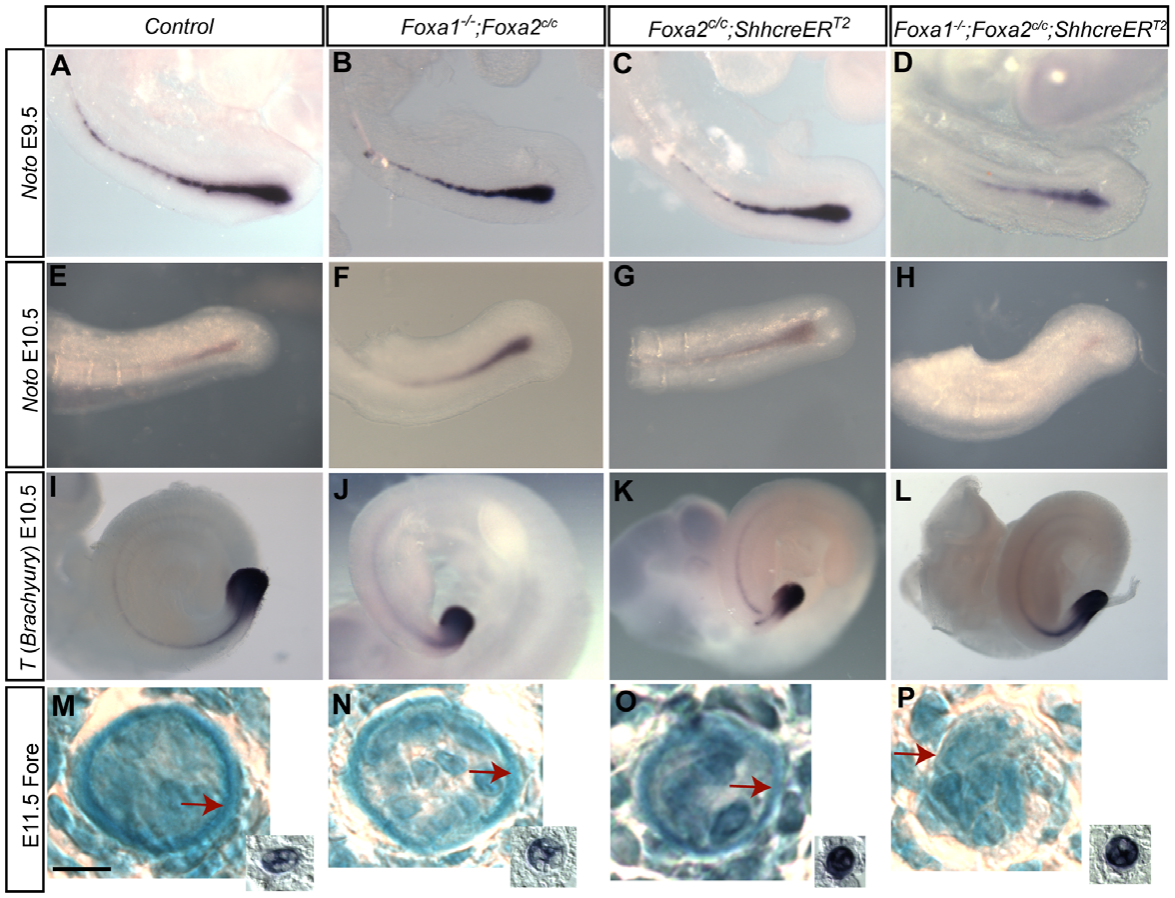
Expression of some notochord genes are unaffected in *Foxa1*;*Foxa2* double knockouts but the notochord sheath is abnormal. A–D: E9.5 and E–H: E10.5 whole-mount *in situ* hybridization for *Noto*. I–L: E10.5 whole-mount *in situ* hybridization for *T* (*Brachyury*). Both genes are expressed in the posterior notochord and expression extends through the tail of the embryo. *Noto* expression at E9.5 in single (B,C) and double mutants (D) was indistinguishable from control embryos (A). However, at E10.5, *Noto* was barely detectable in the tail of double mutants (H). I–L: *T (Brachyury)* expression at E10.5. There was no detectable differences in *T* expression in control (I), single mutant (J,K) and double mutant embryos (L). E11.5 alcian blue and nuclear fast red staining of the notochordal sheath at the forelimb level (M–P). Scale bars are 10 µm. Adjacent or near-adjacent sections of the notochord are pictured in the inset of M–P and show *T (Brachyury) in situ* hybridization of the notochord. At the forelimb level, the notochord sheath is clearly visible as a thin blue ring around the notochord in control (M) and single mutant (N,O) embryos (red arrows). In double mutants, the notochord is visible but the sheath is barely apparent (P, red arrow).

The transcription factors *Noto* and *T* (*Brachyury*) were examined by whole-mount RNA *in situ* hybridization. *Noto* is responsible for the truncate (*tc*) mutation in mice, which affects development of the posterior notochord resulting in the lack of a tail [44]. The zebrafish homologue of *Noto*, *flh* (floating head), results in the absence of the notochord [59]. *Noto* expression is reported to be dependent on the initial expression of both *Foxa2* and *T* [44]. To determine if *Noto* was downstream of *Foxa*, RNA *in situ* hybridization was performed on E9.5 and E10.5 double mutant embryos. At this stage of development, FOXA2 protein is absent in the notochord of mutant embryos (see Figure 1D). In E9.5 double mutants and littermates, *Noto* was robustly expressed throughout the tail (Figure 5A–D). By E10.5 *Noto* was not detectable in the tail of double mutants (Figure 5H) but was found in the tails of control (Figure 5E), *Foxa1* null (Figure 5F), and *Foxa2* notochord knockouts (Figure 5G).

*T* is expressed in the notochord [60], [61] and mice heterozygous for a mutation in this gene have been reported to have shorter tails [62]. In *Foxa2* null embryos the notochord does not form and as a result *T* is not expressed. In controls (Figure 5I), mice that lacked *Foxa2* in the notochord (Figure 5K), *Foxa1* null embryos (Figure 5J), and double mutants (Figure 5L), *T* was expressed throughout the posterior notochord at E10.5. The massive cell death observed in E11.5 embryos precluded an analysis of *T* expression at later stages.

### The notochord sheath is abnormal in double mutants

The notochord sheath, composed of an acellular extracellular matrix, surrounds the notochord the length of the embryo. To examine the notochord sheath, transverse sections were stained with alcian blue [14] and nuclear fast red was used as a counterstain. An *in situ* hybridization for *T (Brachyury)* was also performed on adjacent sections in order to accurately visualize the notochord (insets in Figure 5M–P). In control embryos at the forelimb level (Figure 5M), the notochord sheath was observed to surround the notochord. Single knockout embryos also contained a readily apparent notochord sheath (Figure 5N,O). In contrast, double mutant embryos, which did have a notochord visible at the forelimb level (Figure 5P), contained a much more faint notochord sheath.

### Expression of *Shh* and activation of the hedgehog signaling pathway requires *Foxa* gene expression in the notochord

*Shh* is expressed in the notochord and is required for the formation of IVDs [42], [50], [63], [64]. *Shh* and hedgehog signaling have also recently been demonstrated to be required for post-natal growth and differentiation of the mouse disc [65]. The *Shh* notochord enhancer element has been identified and shown to contain binding sites for FOXA family members [66] suggesting that FOXA proteins directly regulate expression of *Shh* within the notochord. The apparent functional redundancy between FOXA family members and the early lethality observed in *Foxa2* null embryos has precluded a genetic analysis of the role FOXA proteins may play in activation of *Shh* and the hedgehog signaling pathway in the vertebrae notochord.

**Figure 6:**
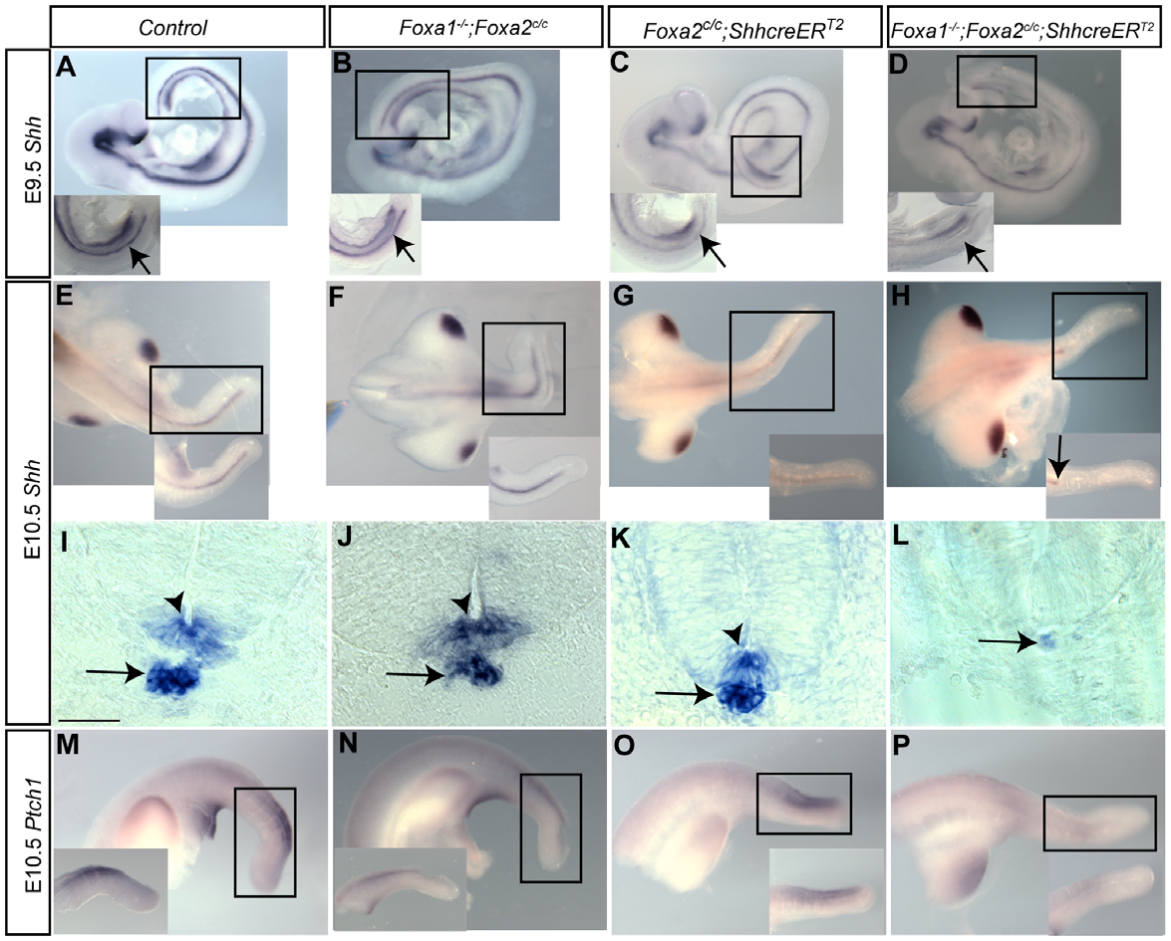
Hedgehog signaling is decreased in *Foxa1*;*Foxa2* double knockouts. A–H: *Shh* whole-mount RNA *in situ* hybridization. At E9.5, *Shh* mRNA is found in the brain, midline, and hindgut of control (A) *Foxa1* null (B) and *Foxa2* knockout embryos (C). In *Foxa1*;*Foxa2* double mutants, midline expression terminates at the posterior end of the embryo (D, arrow) while it remains in control and single knockout embryos (A–C, arrows). At E10.5, *Shh* mRNA was robustly expressed in the posterior limbs and midline of control and single knockout embryos (E, F, and G). In *Foxa1*;*Foxa2* double mutants, *Shh* was expressed in the posterior limbs but absent from the midline of the tail (H). I–L: Scale bars are 50 µm. At the hindlimb level, *Shh* was expressed in the notochord (arrow) and floorplate (arrowhead) in control and single mutant embryos (I, J, and K). No *Shh* expression was observed in the floor plate of E10.5 double mutants (L). Expression was downregulated in the notochord (arrow). I–L are transverse sections at the hindlimb level of E10.5 embryos. M–P: *Ptch1* whole-mount *in situ* hybridization. *Ptch1* expression was decreased in the tail of *Foxa1*;*Foxa2* double mutants (P) compared to control (M), *Foxa1* null (N) and *Foxa2* mutants (O). Insets in A–D, E–H and M–P are close-up views of the boxed region shown in the respective figures.

To analyze the role FOXA proteins may play in regulating *Shh*, E10.5 double mutant embryos were initially analyzed using RNA *in situ* hybridization. In control (Figure 6E,I), *Foxa1* null (Figure 6F,J) or *Foxa2* notochord knockout mice (Figure 6G,K), *Shh* was robustly expressed in the notochord and floorplate . In *Foxa1*;*Foxa2* double knockout embryos, *Shh* expression was decreased in the notochord of the tail and absent from the floorplate (Figure 6H and L). *Shh* was expressed robustly in the posterior limbs of double mutants (Figure 6H), consistent with the absence of CRE expression in these tissues (at E7.5, the time point of tamoxifen exposure, the *ShhcreER^T2^* allele is not expressed in the limbs since these structures have not yet formed). To examine if this decrease of *Shh* occurred earlier, we also performed *in situ* hybridization with E9.5 mice. Control (Figure 6A), *Foxa1* null (Figure 6B), and *Foxa2* notochord knockout (Figure 6C) mice had robust expression of *Shh* in the brain, hindgut, and midline. In contrast, the double mutant embryos lost *Shh* from the midline in the distal half of the body, though it was maintained in the hindgut and brain (Figure 5C, arrow).

To confirm a decrease in hedgehog signaling in the notochord of double mutants, *Ptch1*, a direct target of the hedgehog pathway, was examined [49], [67]. In the tails of double knockout embryos, *Ptch1* was decreased (Figure 6P) compared to control littermates and embryos that had either *Foxa1* or *Foxa2* absent in the notochord (Figure 6M,N, and O).

### *Foxa* expression in the notochord is required for dorsal-ventral patterning of the neural tube

The notochord is an important embryonic signaling center that is required for patterning the floorplate [16]. *Shh* secreted from the notochord patterns the floorplate in a concentration-dependent manner [68]. Since *Shh* was decreased in *Foxa1*;*Foxa2* double mutants (Figure 6), it was possible that neural tube patterning would be aberrant. Furthermore, dorsal ventral mispatterning of the neural tube has been described in *Foxa2* null mice [34], [35].

**Figure 7:**
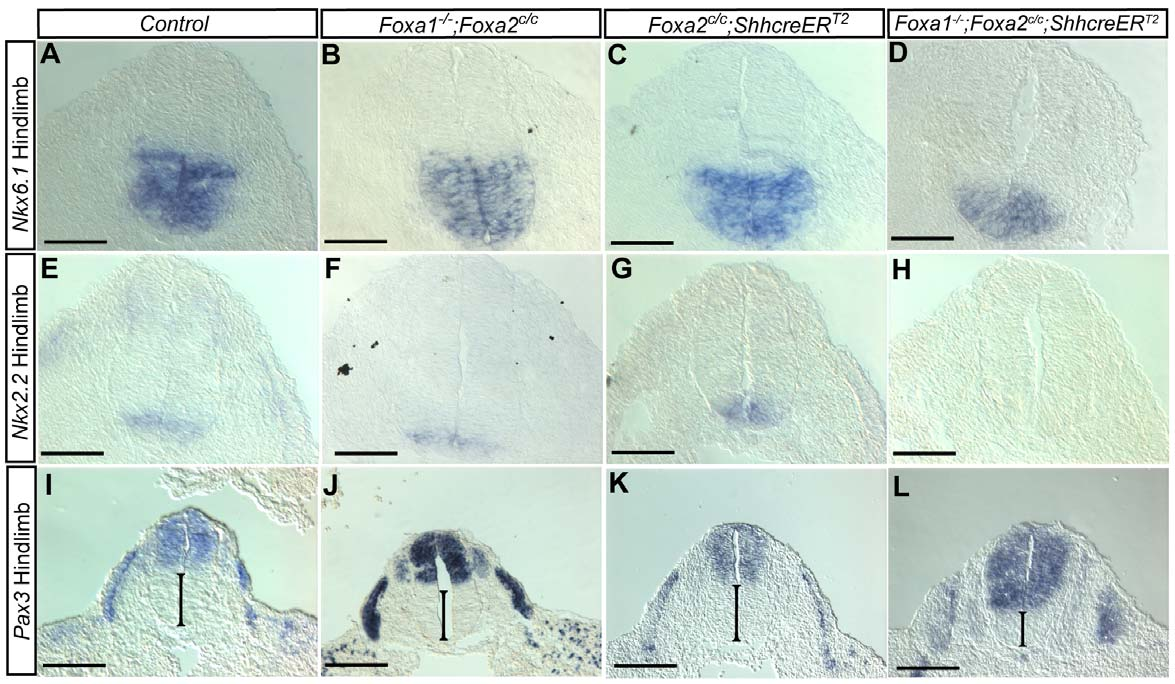
Dorsal-ventral patterning of the neural tube is abnormal in *Foxa1*;*Foxa2* double knockouts. Section *in situ* hybridization at E10.5 for *Nkx6.1* (A–D). Control (A), *Foxa1* nulls (B) and *Foxa2* notochord knockout (C) embryos had similar expression patterns in the ventral neural tube. However, in double *Foxa1*;*Foxa2* knockouts (D), *Nkx6.1* mRNA was restricted to a ‘wedge’ shape at the bottom of the neural tube. E–H: *Nkx2.2* was constricted to a ventrally-located band of cells in the neural tube in controls (E), *Foxa1* nulls (F) and *Foxa2* notochord knockout (G) embryos. In *Foxa1*;*Foxa2* double mutants, *Nkx2.2* was undetectable (H). I–L: *Pax3* is normally expressed in the dorsal neural tube and in the dermomyotome. In control (I), *Foxa1* nulls (J) and *Foxa2* notochord knockouts (K) *Pax3* was expressed normally. In double mutants (L), *Pax3* expression was expanded ventrally (black bar). *Pax3* expression in the dermomyotome of double mutants was normal (L). A–H: Scale bar 100 µm, I–L: Scale bar 200 µm.

*Nkx6.1*, a transcription factor that is typically expressed in the ventral half of the neural tube [48], was found to be normal at the hindlimb level of control (Figure 7A) *Foxa1* null (Figure 7B) and *Foxa2* notochord knockout (Figure 7C) embryos at E10.5. In double mutants, *Nkx6.1* expression was restricted to a smaller wedge-shaped area in the double mutant when compared to its littermates (Figure 7D). *Nkx2.2* is normally expressed on either side of the neural tube just above the floorplate. In double mutants (Figure 7H) expression was undetectable at the hindlimb level. Expression was present in the ventral neural tube of controls and single mutants (Figure 7E, F and G). *Pax3*, which is expressed in the dermomyotome and dorsal neural tube [47], was examined in E10.5 embryos (Figure 7I–L). At the hindlimb level, *Pax3* was extended ventrally in the neural tube of *Foxa1*;*Foxa2* double mutants (Figure 7L). Dermomyotome expression of *Pax3* appeared unchanged in double mutants. At the level of the double mutant forelimb, expression of all genes examined was indistinguishable from controls (Figure S3 and File S1).

## Discussion

### *Foxa1* and *Foxa2* are functionally redundant in the notochord

*Foxa2* null mice die lacking the node, which during later embryonic development forms the notochord [34], [35]. To bypass the early requirement for *Foxa2* in node formation, we removed *Foxa2* from the notochord using a tissue-specific *Cre* line. In these mice, *Foxa2* was expressed in the node but not the notochord, which allowed for the characterization of the role this gene played in the notochord and disc development. In *Foxa1*;*Foxa2* double mutants, *Foxa1* was absent from all cells (null allele) while *Foxa2* was only removed from the notochord in the vertebral column. Since *Foxa1* null mice die postnatally and have fully-formed discs ([26] and this report) the phenotypes present in the double mutants were likely due to the removal of both of these genes from the notochord. However, we cannot rule out the possibility that *Foxa2* expression in the floorplate plays a role in disc development in vertebral columns that lack *Foxa2* in the notochord. In our study, *Foxa2* was not removed from the floorplate since the *Shh* promoter was not active in the floorplate during the time that embryos were exposed to tamoxifen.

### The caudal vertebral column is more severely affected in *Foxa1*;*Foxa2* double knockouts

A common feature present in most mouse mutants that have abnormal notochord development is that the caudal part of the animal is more severely malformed than the rostral part [44], [61], [63]. Consistent with these reports, the tails of double mutant *Foxa1*;*Foxa2* mice lacked any discernable vertebrae or disc structures at birth. The only exception was two animals that had shortened tails with discs in them, but thoraco-lumbar discs were compressed and small. It is possible that in these animals that tamoxifen was administration at slightly later stages since plug dates are only estimated. In double mutant animals, defects were less severe in the rostral region of the embryo with well-developed discs found above the forelimb level in double mutants.

The forming notochord has been fate mapped and found to be composed of three distinct regions: head notochord which is not derived from node cells, trunk notochord that is node-derived, and tail notochord that forms from migrating node cells [19]. The increasing severity of notochord defects in the caudal region of the embryo may be due to the notochord being derived from different regions of the embryo. The *ShhcreER^T2^* allele used in the current study to remove *Foxa2* is expressed throughout the notochord [3]. However, it is currently unknown if this *Cre* allele is expressed in all regions of the notochord at E7.5, the time that tamoxifen was administered to remove *Foxa*2. It is possible that CRE protein driven from the *ShhcreER^T2^* allele is first expressed in caudal notochord cells, which would result in a transient burst of FOXA2 protein expression in the anterior notochord prior to CRE-induced recombination of the *Foxa2* allele.

Despite the fact that there is no tail or posterior notochord at E7.5, when we administer tamoxifen, defects were observed in the tail. This phenotype could be due to knockout of *Foxa2* (and lack of *Foxa1*) in the cells of the node/notochord that are normally supposed to produce this tissue. *Foxa2* will be active for a short period of time before tamoxifen can inactivate this gene. It is possible that a short burst of *Foxa2* transcription is sufficient to begin the formation of the posterior notochord, but the subsequent failure of hedgehog signaling in these cells may prevent the rest of the notochord from forming normally.

### *Foxa1* and *Foxa2* are required for activation of the hedgehog signaling pathway in the notochord

SHH protein secreted from the notochord is required to pattern the neural tube, for formation of the notochordal sheath that surrounds the notochord and formation of nuclei pulposi [15], [42], [50], [63], [64]. In post-natal mice, SHH from the NP is also required for disc differentiation and growth [65]. In *Foxa1*;*Foxa2* double knockouts, *Shh* mRNA was undetectable in the tail by E9.5 and decreased in the notochord at the hindlimb level of E10.5 embryos. *Foxa2* has been proposed to activate transcription of *Shh* in the notochord [34], [35] and other embryonic tissues [29], [37] suggesting that FOXA proteins directly regulate the transcription of *Shh* in the embryonic notochord.

A previous report identified a *Shh* notochord enhancer that was sufficient to drive reporter expression specifically in the notochord [66]. This enhancer element contained three *Foxa* binding sites that were required for reporter gene expression in the notochord [66]. However, due to the early lethality of *Foxa2* null embryos and functional redundancy between FOXA proteins, previous studies have been unable to confirm *Shh* regulation by FOXA proteins *in vivo*. Our data directly demonstrate that expression of *Shh* in the notochord requires FOXA proteins, suggesting that the previously identified FOXA binding sites in the *Shh* notochord enhancer are functional. Consistent with these data *Ptch1* expression, a direct target of hedgehog signaling, in the caudal region of *Foxa1*;*Foxa2* double knockouts was also reduced.

The observed defects in the notochord sheath in double mutants are also consistent with a decrease in hedgehog signaling [63]. The lack of a sheath to contain cells within the notochord in double mutants may cause the observed dispersal of LacZ-stained notochord cells in our fate-mapping experiment. Without the notochordal sheath to maintain cells within the notochord these cells may be pushed to ectopic locations during formation of the vertebral column.

The phenotype of the *Foxa1*;*Foxa2* double mutant mouse described in this report is similar to mice that we previously characterized in which hedgehog signaling was removed from both the notochord and floorplate [63]. In our previous report, *Smoothened* was removed and disc development assessed. The NP was found to be small and compressed, and the notochord to nucleus pulposus transition was also abnormal. Choi et al did not observe the dorso-ventral mispatterning of the neural tube observed in *Foxa1*;*Foxa2* double mutants, possibly because *Shh* is still being expressed from the notochord in mice that lacked *Smoothened* from the notochord. We have also previously shown that removal of *Shh* from the floorplate had no affect on disc and vertebral development, while removal of *Shh* from the notochord resulted in a severe deformation of the entire vertebral column and decreased *Pax1* expression at E11.5 [50].

### The neural tube was “dorsalized” in *Foxa1*;*Foxa2* double mutants

Studies using explant cultures have shown that *Shh* signaling counteracts expression of genes that are required for formation of the dorsal neural tube [69]. The expansion of dorsally expressed genes in the neural tube and the loss of ventral neural tube identity and motor neurons have been documented in the *Foxa2* null mouse [34], [35]. A similar phenotype has also been reported in the *Shh* null mouse [64]. In mice in which *Foxa2* was absent in all cells (null animals), *Pax3* was expanded throughout the entire neural tube [34] while markers of motor neurons such as *Islet-1* were reported to be absent from the neural tube [35].

In double *Foxa1*;*Foxa2* mutants, in which *Foxa2* was absent from the notochord, expression domains of dorsal neural tube genes were expanded at the expense of ventrally expressed genes (a “dorsalized” neural tube). The loss of dorsal-ventral identity in the neural tube of double *Foxa1*;*Foxa2* mutants was consistent with the observed decrease in *Shh* expression in the notochord.

In the caudal region of double *Foxa1*;*Foxa2* mutants, the floorplate appeared to be absent. The *Foxa2* null mouse has also been reported to lack a floorplate (presumably because no notochord forms in null embryos resulting in the absence of notochord-produced SHH protein) [34], [35]. We speculate that the floorplate in *Foxa1*;*Foxa2* double mutants does not form in the caudal embryo due to a requirement of FOXA expression in the notochord, which is required to activate *Shh* [68].

### *Noto* expression is not maintained in *Foxa1*;*Foxa2* double knockout embryos

*Noto* has been proposed to be a target of FOXA2 since it is not expressed in chimeras between *Foxa2* null ES cells and wild-type tetraploid embryos [44]. Tetraploid chimeras between *Foxa2* null ES cells and wild-type embryos have been described, and while they do not form the node or notochord, primitive streak morphogenesis is rescued [70]. In our experiments, *Noto* was robustly expressed in the notochord and node of all double mutant embryos examined at E9.5. However at E10.5, *Noto* was absent from the tail of double knockout embryos. The presence of a notochord in E10.5 embryos was confirmed by analysis of *T*, which is expressed in the caudal notochord. These data suggest that *Noto* requires FOXA in the notochord to maintain its expression. However, it is currently unclear if FOXA proteins directly regulate *Noto* expression or if transcription of this gene is downstream of the hedgehog signaling, or a yet unidentified signaling pathway.

It is likely that other pathways besides hedgehog signaling are responsible for disc formation. Removal of hedgehog signaling from the post-natal disc both *in vivo* and *in vitro* was reported to decrease TGF-β and Wnt signaling [65]. In addition, Wnt signaling was shown to be required for posterior tail development. Early in development though, *Foxa1* and *Foxa2* are both still expressed in the β-catenin knockout notochord [20]. Finally, a recent screen of *Foxa2* targets in the notochord identified seven genes that were previously not recognized as *Foxa2* targets expressed in the node, posterior notochord, and throughout the entire notochord [71]. It is possible that one or more of these genes may be downregulated in *Foxa1*;*Foxa2* double knockout.

Our data demonstrates that both *Foxa1* and *Foxa2* are required for disc formation. The loss of both *Foxa1* and *Foxa2* from notochord cells results in an aberrant notochord to NP transition, cell death in the posterior somites and tail, and severe deformation of the NP. Our data provide *in vivo* evidence for *Shh* being a direct downstream target of the *Foxa* signaling pathway in the notochord suggesting that the previously identified *Foxa* binding sites in the *Shh* notochord enhancer are functional.

## Supporting Information

Figure S1 *Foxa1* and *Foxa2* expression in the notochord. A–D: Whole-mount *Foxa1 in situ* hybridization. I–K: Section *in situ* hybridization for *Foxa1*. *Foxa1* is undetectable at E7.5 (A), but expressed in the notochord at E8.5 (B–D,I,J), floorplate (B–D, J), midbrain and gut (B–D, I). It remains on in the notochord until E12.5 (not shown). *Foxa1* is not expressed in the NP at E14.5 (K). E–H: Whole-mount *Foxa2 in situ* hybridization. L–N: Section *in situ* hybridization for *Foxa2*. *Foxa2* is detectable at E7.5 (E) and remains faintly in the notochord until E8.5 (F,L). It is also expressed in the floorplate, gut, and midbrain (F–H). *Foxa2* remains detectable in the floorplate until at least E14.5 (F–H, L–M, and data not shown). Purple staining in brain vesicles in (G) is signal pooling in the brain. Scale bars are 50 µm for I and L, 100 µm for J and M, and 200 µm for K and N. Black arrowhead in I,J,L and M point to the notochord.
(TIF)

Figure S2 Vertebral abnormalities and shortened tail in double mutant. A–D: Picrosirius red and Alcian blue staining of E19.5 embryos (*Foxa1* null is E17.5). Control (A), *Foxa1* null (B) and *Foxa2* notochord knockout (C) ossification centers within the vertebral bodies are indistinguishable from one another. Double knockout vertebral column showed a split ossification center (D). Scale bars: A–D: 400 µm. E–G: Picrosirius red and Alcian blue stained tail sections. Nucleus pulposus tissue is easily identifiable in control (E), and single mutant (F and G) embryos at E17.5. In double mutants, the tail was shortened and frequently lacked identifiable discs and vertebrae (H). Scale bars: E–H 100 µm. I: E17.5 embryos, left: control littermate, right: *Foxa1*;*Foxa2* double mutant showing a drastically shortened tail.
(TIF)

Figure S3 Forelimb-level expression of sclerotome and neural tube markers are normal in double mutants. A–D: Forelimb level sections of *Pax1*. Controls (A), *Foxa1* null (B) and *Foxa2* notochord knockouts (C) and double *Foxa1*;*Foxa2* knockouts (D) have robust *Pax1* staining in the sclerotome. E–H: Forelimb level sections of *Nkx6.1*. *Nkx6.1* is confined to the ventral half of the neural tube in control (E), *Foxa1* null (F), *Foxa2* notochord knockout (G), and double *Foxa1*;*Foxa2* knockout embryos (H). I–L: *Nkx2.2* at the forelimb level. *Nkx2.2* is expressed just dorsal to the floorplate in control (I), *Foxa1* null (J), *Foxa2* notochord knockout (K), and double *Foxa1*;*Foxa2* knockouts (L). M–P: *Pax3* at forelimb level. *Pax3* is expressed in the dermomyotome and dorsal half of the neural tube in control (M), *Foxa1* null (N), *Foxa2* notochord knockout (O), and double *Foxa1*;*Foxa2* knockouts (P). Scale bars: A–D, M–P: 200 µm. E–L: 100 µm.
(TIF)

File S1 Unabridged in situ protocol.
(DOC)
