# Supplementary figures and images for: Foxa1 and Foxa2 Are Required for Formation of the Intervertebral Discs
Source: PLoS One. 2013 Jan 31;8(1):e55528. doi: 10.1371/journal.pone.0055528 (PMC3561292; doi:10.1371/journal.pone.0055528)

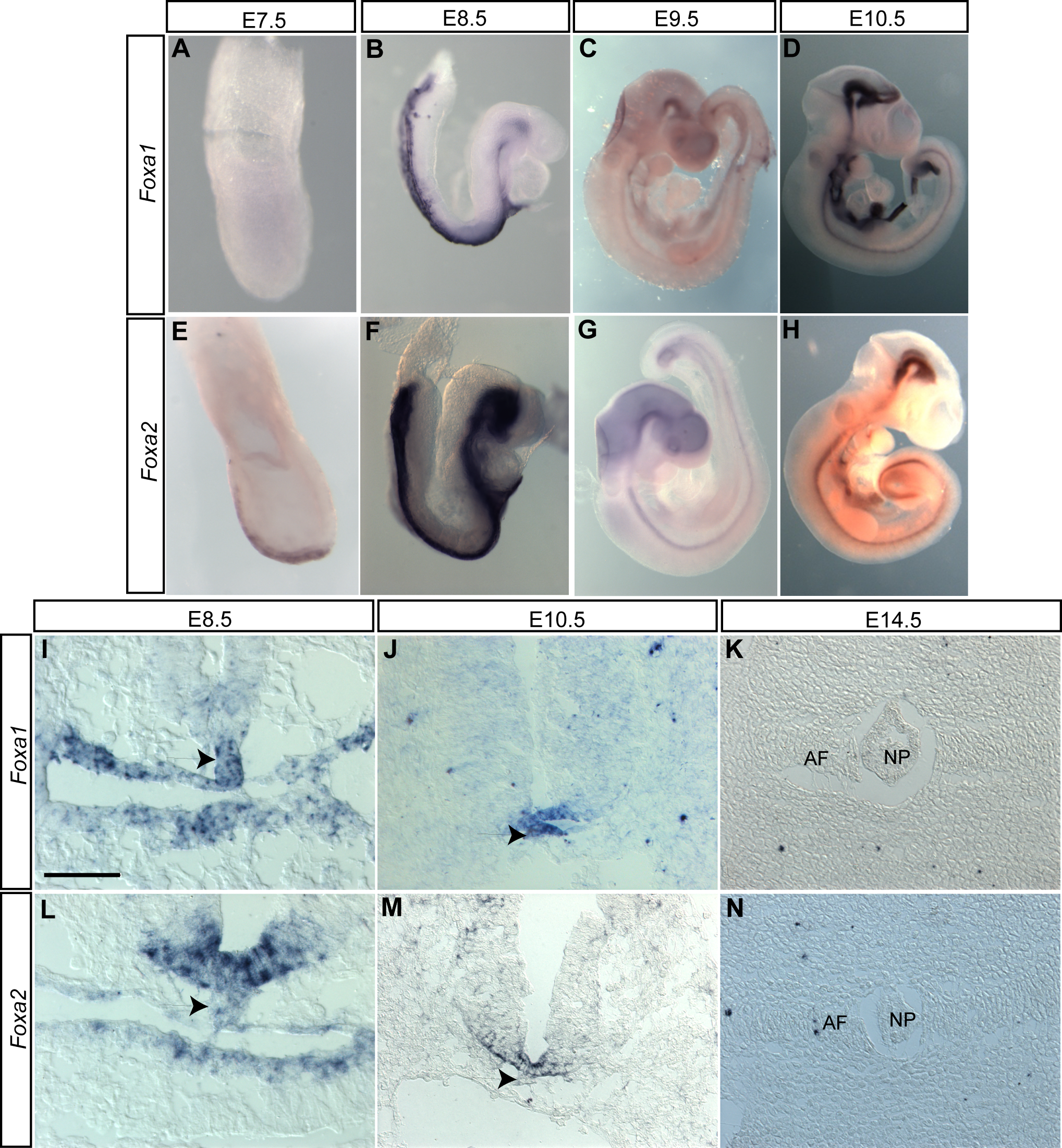

Supplement: Figure S1 — Foxa1 and Foxa2 expression in the notochord. A–D: Whole-mount Foxa1 in situ hybridization. I–K: Section in situ hybridization for Foxa1. Foxa1 is undetectable at E7.5 (A), but expressed in the notochord at E8.5 (B–D,I,J), floorplate (B–D, J), midbrain and gut (B–D, I). It remains on in the notochord until E12.5 (not shown). Foxa1 is not expressed in the NP at E14.5 (K). E–H: Whole-mount Foxa2 in situ hybridization. L–N: Section in situ hybridization for Foxa2. Foxa2 is detectable at E7.5 (E) and remains faintly in the notochord until E8.5 (F,L). It is also expressed in the floorplate, gut, and midbrain (F–H). Foxa2 remains detectable in the floorplate until at least E14.5 (F–H, L–M, and data not shown). Purple staining in brain vesicles in (G) is signal pooling in the brain. Scale bars are 50 µm for I and L, 100 µm for J and M, and 200 µm for K and N. Black arrowhead in I,J,L and M point to the notochord. (TIF) [file pone.0055528.s001.tif]

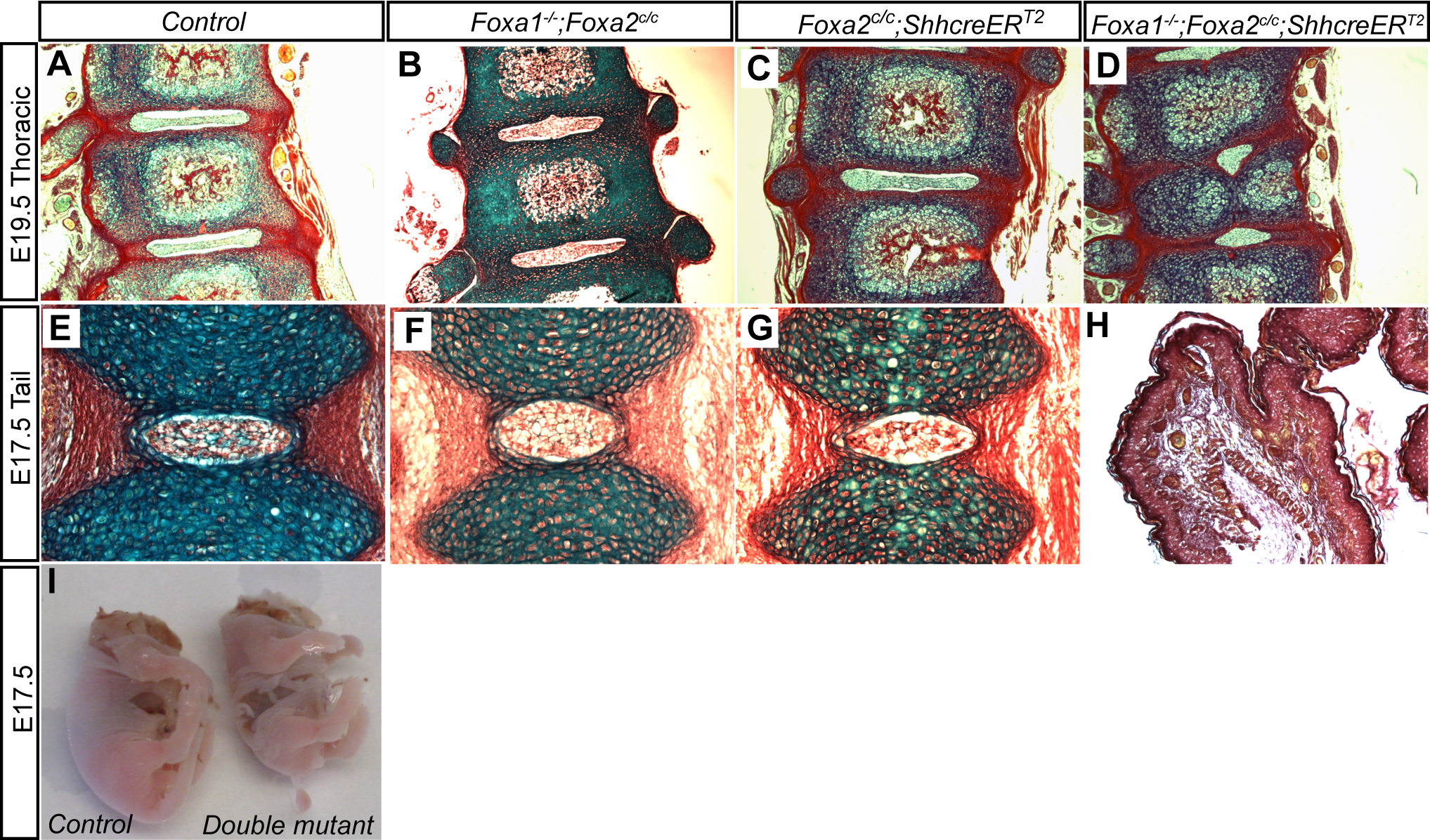

Supplement: Figure S2 — Vertebral abnormalities and shortened tail in double mutant. A–D: Picrosirius red and Alcian blue staining of E19.5 embryos (Foxa1 null is E17.5). Control (A), Foxa1 null (B) and Foxa2 notochord knockout (C) ossification centers within the vertebral bodies are indistinguishable from one another. Double knockout vertebral column showed a split ossification center (D). Scale bars: A–D: 400 µm. E–G: Picrosirius red and Alcian blue stained tail sections. Nucleus pulposus tissue is easily identifiable in control (E), and single mutant (F and G) embryos at E17.5. In double mutants, the tail was shortened and frequently lacked identifiable discs and vertebrae (H). Scale bars: E–H 100 µm. I: E17.5 embryos, left: control littermate, right: Foxa1;Foxa2 double mutant showing a drastically shortened tail. (TIF) [file pone.0055528.s002.tif]

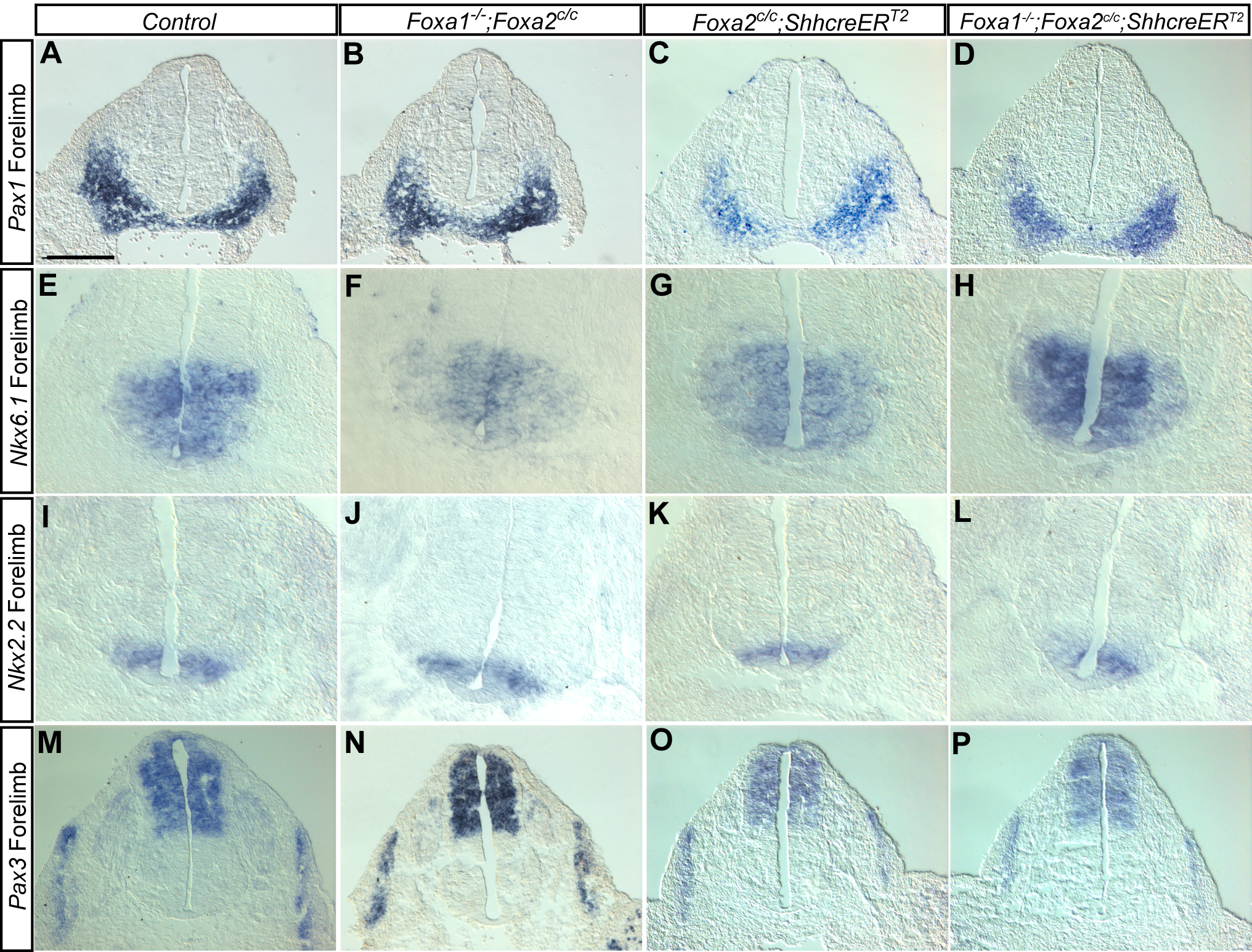

Supplement: Figure S3 — Forelimb-level expression of sclerotome and neural tube markers are normal in double mutants. A–D: Forelimb level sections of Pax1. Controls (A), Foxa1 null (B) and Foxa2 notochord knockouts (C) and double Foxa1;Foxa2 knockouts (D) have robust Pax1 staining in the sclerotome. E–H: Forelimb level sections of Nkx6.1. Nkx6.1 is confined to the ventral half of the neural tube in control (E), Foxa1 null (F), Foxa2 notochord knockout (G), and double Foxa1;Foxa2 knockout embryos (H). I–L: Nkx2.2 at the forelimb level. Nkx2.2 is expressed just dorsal to the floorplate in control (I), Foxa1 null (J), Foxa2 notochord knockout (K), and double Foxa1;Foxa2 knockouts (L). M–P: Pax3 at forelimb level. Pax3 is expressed in the dermomyotome and dorsal half of the neural tube in control (M), Foxa1 null (N), Foxa2 notochord knockout (O), and double Foxa1;Foxa2 knockouts (P). Scale bars: A–D, M–P: 200 µm. E–L: 100 µm. (TIF) [file pone.0055528.s003.tif]
