# Supplementary material for: Foxa1 and Foxa2 Are Required for Formation of the Intervertebral Discs
Source: PLoS One. 2013 Jan 31;8(1):e55528. doi: 10.1371/journal.pone.0055528 (PMC3561292; doi:10.1371/journal.pone.0055528)
Supplement: File S1 — Unabridged in situ protocol. (DOC) [file pone.0055528.s004.doc]

**Supporting Information**

***Unabridged in situ protocol***

***-*Whole Mount *in situ* hybridization**

Day 1

__Fix harvested embryos in 4% PFA overnight

__PBT 2 x 5 min

__MeOH series: 5 min each in 25%, 50%, 75%, 100% MeOH in PBT

__1 hour in 6% H_2_O_2_ diluted in 100% MeOH

__5 min 100% MeOH

__Rehydrate: 10 min each in 75%, 50% and 25% MeOH

__3 x 5 min PBT

__Proteinase K treatment (dilute in PBT): E7.5/E8.5: No Prot K treatment, just go to fix, E9.5: 10mg/mL for 10 min. E10.5: 10mg/mL for 15-20 min

__Rinse in PBT, put in 4% PFA/0.2% glutaraldehyde/PBT for 20 min

__3 x 5 min PBT

__Wash in prehybridization solution for at least 1 hour at 70ºC; Change prehybridization solution and add probe (1µL), incubate overnight

Day 2:

__Wash with Solution 1, 4 x 30 min 70ºC

__Rinse in 50% Solution 1: 50% MABT 5 min room temperature

__2 x 30 min in MABT

__1 hr in 2% Blocking reagent/MABT

__1-2 hrs in 2% Blocking reagent/20% Heat-inactivated goat serum/MABT

__Add antibogy 1:2000 in fresh blocking solution with serum, incubate overnight 4ºC

Day 3: Wash all day in MABT (at least 5-6 changes)

Day 4:

__4x 10 min NTMT

__Add BM Purple (Roche) to cover embryos, wrap in foil, develop until color comes up, rinse several times in NTMT, then PBT, fix overnight at 4ºC and store in dark.

**Solutions for whole-mount *in situ***:

1x PBT: 1x PBS + 0.1% Tween-20

Prehybridization solution: 50% formamide. 5x SSC (pH4.5), 2% SDS, 2% BR (Blocking reagent, Roche), 250μg/mL tRNA (Roche), 100μg/mL heparin (Sigma)

Mix dry ingredients, add liquids, use water to bring to volume, mix and dissolve at 65ºC until blocking reagent is in solution, aliquot and freeze at -20ºC.

Solution 1: 50% formamide, 2x SSC pH4.5, 1% SDS, bring to volume with water.

1x MABT: 100mM maleic acid, 150mM NaCl, bring to volume with water. pH with NaOH pellets to pH7.5. Add Tween-20 to 0.1%.

1x NTMT: 100mM NaCl, 100mM Tris pH9.5, 50mM MgCl_2_, 0.1% Tween-20.

**-Section *in situ* hybridization:**

Day 1: (Use DEPC-solutions)

__Thaw sections in a slide box

__Trace around sections with hydrophobic pen

__Wash with water

__15 min 0.12M HCl

__2 x 5 min PBS

__Proteinase K (1µg/mL) in PBS, 6 min

__10 min PBS

__Fix 5 min (4% PFA)

__2 x 5 min PBS

__0.25% acetic acid 10 min

__2 x 5 min PBS

__Add prehybridization solution with 10% dextran sulfate to slides. At same time incubate probe in solution (1µL/slide) at 65ºC for 15 min

__Add probe in prehybe to slides, cover with parafilm strips, incubate overnight at 65ºC

Day 2: Pre-warm solutions, remove coverslips

__5 min 5x SSC + 0.1% Triton X100, 65ºC

__30 min 2x SSC + 50% formamide + 0.1% Triton X100, 65ºC

__2 x 30 min 2x SSC + 0.1% Triton X100, 65ºC

__2 x 30 min 0.2x SSC + 0.1% Triton X100, 65ºC

__Rinse in KTBT at room temperature 3 times

__Block in 10% goat serum (heat inactivated)/KTBT 1 hr. Also block antibody in 5% goat serum/KTBT (1:2000)

__Add antibody, coverslip with parafilm, incubate overnight at 4ºC

Day 3: Wash all day in KTBT, leave at 4ºC overnight

Day 4: Wash in AP buffer 2 x 20 min

Add BM Purple (Roche) to slides, coverslip with parafilm, incubate at 37ºC until color comes up

Wash in AP buffer, rinse in PBS, fix in 4% PFA, rinse in PBS and mount with Glycergel (DAKO).

**Solutions for section *in situ***:

Prehybridization: Same as for whole-mount, but dissolve dextran sulfate to 10%

1x KTBT: 50mM Tris pH7.5, 150mM NaCl, 10mM KCl, 0.1% Tween-20

1x AP: 100mM NaCl, 100mM Tris pH9.5, 0.1% Tween-20

***Foxa1 and Foxa2 are expressed in the notochord and nuclei pulposi***

To characterize in detail the expression patterns of *Foxa1* and *Foxa2*, section and whole-mount RNA *in situ* hybridizations were performed on wild-type and control embryos from E7.5 through E14.5 using anti-sense DIG-labeled probe (Figure S1). *Foxa1* (Figure S1A-D, I-K) and *Foxa2* (Figure S1E-H, L-N) were found to have very similar expression patterns consistent with previous reports . *Foxa1* was expressed in the notochord from E8.5 (Figure S1B, I) through E12.5 (Figure S1C, D, I and J and data not shown). *Foxa1* was also detectable in the floorplate at all stages through E14.5 (Figure S1C,D,J and data not shown). Our studies indicate that *Foxa1* was not expressed in nuclei pulposi or in any other disc structure (Figure S1K). Consistent with a previous report , *Foxa1* was not detected at E7.5 (Figure S1A).

*Foxa2* has been reported to be expressed in similar tissues as *Foxa1*, though it is reported to be expressed earlier (E6.5) in the primitive streak . *Foxa2* was expressed in the node and notochord at E7.5 and remained faintly detectable in the notochord at E8.5 (Figure S1 E,F and L). By E9.5 and onwards expression was absent in the notochord (Figure S1M and data not shown). Robust expression in the floorplate was detected at E8.5 and continued to be expressed in this tissue until at least E12.5 (Figure S1 F-H,L,M and data not shown). *Foxa2* was not expressed in the forming disc (Figure S1N). *Foxa1* and *Foxa2* expression were also found in endoderm and midbrain tissues, consistent with previous reports .

***Ossification centers are bifurcated and the tail is shortened in Foxa1;Foxa2 double mutants***

Vertebral abnormalities were present in *Foxa1;Foxa2* double mutant animals. Histological sections show a split ossification center (Figure S2C) in double mutant vertebral columns in addition to a deformed NP. Control (Figure S2A) and *Foxa2* notochord knockout (Figure S2B) animals are indistinguishable from one another. A shortened tail, described in the main text, is observed in double mutants (Figure S2I).

***Sclerotome and neural tube gene expression is unchanged at the forelimb level of Foxa1;Foxa2 double mutants***

*Pax1*, which was unchanged in hindlimb sections of E10.5 double mutant embryos, is also expressed in the same pattern in the sclerotome at the forelimb level (Figure S3A-C). All neural tube markers (*Nkx2.2*, *Nkx6.1*, and *Pax3*), which were aberrantly expressed in the double mutant embryo at the hindlimb level, were indistinguishable from their littermates (Figure S3D-L) at the forelimb level at E10.5.

**References**

1. Monaghan AP, Kaestner KH, Grau E, Schutz G (1993) Postimplantation expression patterns indicate a role for the mouse forkhead/HNF-3 alpha, beta and gamma genes in determination of the definitive endoderm, chordamesoderm and neuroectoderm. Development 119: 567-578.

2. Kaestner KH, Hiemisch H, Luckow B, Schutz G (1994) The HNF-3 gene family of transcription factors in mice: gene structure, cDNA sequence, and mRNA distribution. Genomics 20: 377-385.

3. Sasaki H, Hogan BL (1993) Differential expression of multiple fork head related genes during gastrulation and axial pattern formation in the mouse embryo. Development 118: 47-59.
